# Supplementary material for: Lipid-protein interactions modulate the conformational equilibrium of a potassium channel
Source: Nat Commun. 2020 May 1;11:2162. doi: 10.1038/s41467-020-15741-8 (PMC7195391; doi:10.1038/s41467-020-15741-8)
Supplement: Supplementary file 1 — Supplementary Information for Lipid–Protein Interactions Modulate Conformational Equilibrium of a Potassium Channel [file 41467_2020_15741_MOESM1_ESM.pdf]

Supporting Information for

Lipid-Protein Interactions Modulate Conformational Equilibrium of a Potassium Channel

Authors: Gu et al.

This PDF includes:

Supplementary Notes 1-7

Supplementary Methods

Supplementary Tables 1-7

Supplementary Figures 1-13

Supplementary References 1-40

Supplementary Note 1 - Effects of lipid-protein interactions on the activity of potassium channels reported in literature.

The structure and function of potassium channels are known to be sensitive to the membrane environment. For instance, phosphatidylinositol 4, 5-bisphosphate (PIP2), a negatively charged lipid, modulates the gating of potassium channels (e.g., the inwardly rectifying potassium channel GIRK, and Kir, and the voltage gated potassium channel Kv) by specific lipid-protein interactions.[1–6] Valiyaveetil *et al.*[7] showed that potassium conduction of KcsA depends on the presence of negatively charged phosphatidylglycerol lipids (PG). In addition, specific binding sites of cholesterol were identified on the protein surface of GIRK and Kir channels.[8–10] In terms of general membrane properties, negative charges on the membrane surface accelerate potassium permeation by polarizing their local environment in combination with an increasing ion concentration[11–13] at the water-membrane interface. Experimental studies[14–17] revealed lipid-dependent gating of the voltage gated potassium channels, which was ascribed to interactions between the voltage sensing domains and the phospholipid headgroups, in particular the anionic phosphate groups. Therefore, the lipid composition (i.e., concentration of lipids with anionic phosphate groups) exerts strong effects on the equilibrium between its resting and its activated state.[18,19] Lateral pressure also modulates the open probability and ion conduction rate of potassium channels. One example is formed by members of the two-pore domain potassium channel (K2P) family, which are gated by membrane lateral pressure.[20,21] In addition, larger lateral stress in thicker membranes is observed to reduce the open probability and increase the energy barrier of conformational changes between the open and closed states of potassium channels such as the large-conductance Ca^{2+}-activated K^+ channel (BK_{Ca})[22,23] and KcsA[24].

Supplementary Note 2 - Effects of the outer helix on ion permeation rate revealed by restrained simulations.

Restrained simulations confirm the correlation between the conformation of the outer helix and the ion permeation rate. We observe a larger Pro19 Cα-Cα distance of 3.5 nm accompanying an optimum current in POPC, and a smaller Pro19 Cα-Cα distance of 3.2 nm with an almost closed channel in DEPC (Fig. 3C, F). Restraining the Pro19 Cα-Cα distance in POPC to 3.2 nm reduces ion current from 6.3 pA to 2 pA, whereas restraining it to 3.5 nm in DEPC increases the ion conduction rate from 1 pA to 4.7 pA (Supplementary Table 4).

Supplementary Note 3 - Coupling between the conformations of the inner and outer helices.

Both restrained and unrestrained simulations support the coupling between the conformations of the two helices. Smaller Pro19 Cα-Cα distances roughly correlate with a lower fraction of the bent state of the inner helix in simulations using different membranes (Fig. 3G, Supplementary Table 2). In addition, as aforementioned, we divide each of the DLPC, DYPC, DOPC, and DPPC systems in two groups, and the Pro19 Cα-Cα distances are smaller for the groups with a lower average fraction of the bent sate (Supplementary Table 3 and Supplementary Fig. 1). Moreover, a restraint on the conformation of one helix changes the conformation of the other: e.g., restraining the Pro19 Cα-Cα distance to a larger value induces a smaller bending angle (Supplementary Fig. 2) and a higher bent state fraction in DEPC (8% v.s. 1% in unrestrained simulations, Supplementary Table 4), whereas restraining all four subunits in their kinked and bent state results in smaller and larger Pro19 Cα-Cα distances, respectively (Supplementary Table 4 and Supplementary Fig. 1). In addition, functional mode analysis (FMA) indicates a correlation of a larger bending angle of the inner helix (kinked state) with the clockwise rotation of the outer helix, which reduces the Pro19 Cα-Cα distance (Fig. 4A). We ascribe the coupling of these two helices to hydrophobic contacts between residue side chains from these two helices, including interactions between Ile23 and Val89, and the interactions between Leu24 and Leu94.

However, we note that coupling of the conformations of the two helices only partially explain how the outer helix affects ion permeation. In the aforementioned POPC simulation with the Pro19 Cα-Cα distance restrained to a smaller value, three subunits of the tetramer are stabilized in the kinked state, but the remaining one was trapped in the initial conformation, the bent state (Supplementary Fig. 2). In this case, the reduced current may not be fully ascribed to stabilization of the kinked state of the inner helix, and the motion of the outer helix may participate in regulating ion conduction via other mechanisms. Also, the correlation between the conformations of two helices is not perfect (see Fig. 3G and Supplementary Fig. 1), suggesting other reasons that may stabilize the kinked state of the inner helix, in addition to the clockwise rotation of the outer helix. One exception is the DLPC simulation, in which the larger bending of the inner helix is induced by the thickness mismatch between the hydrophobic region of the

protein and the bilayer, and in that case, a coupling of the conformations of the two helices is not observed.

Supplementary Note 4 - Conformational changes across the selectivity filter.

We compared the "free energy" profiles of potassium along the permeation pathway in simulations using different membranes (three bilayers with different ion currents are shown as examples in Fig. 4C). The higher energy barriers for potassium transfer between different binding sites in the selectivity filter (Fig. 4C) in low conductive simulations suggest involvement of all four potassium binding sites in ion permeation rate and conformational changes across the selectivity filter. The Thr59 Oγ-Oγ distance and the Thr59 and Gly61 backbone oxygen distances (Fig. 4D) correlate with each other to a certain extent, supporting a cooperative motion of the selectivity filter residues. Therefore, the selectivity filter opening is not only facilitated by the local expansion of the intracellular entrance (determined by Thr59 Oγ-Oγ distance), but also a global motion of the whole selectivity filter.

In addition, we observe a correlation between the Thr59 Oγ-Oγ distance (as well as the Ile84-Thr59 side chain distance) and the potassium occupancies at S4 and S3 sites (Supplementary Fig. 5): A larger Oγ-Oγ distance results in lower occupancy at S4 site and a higher occupancy at S3 site. This observation indicates that opening degree of the selectivity filter not only affects the energy barriers for ion transition between different potassium binding sites (Fig. 4C), but also modulates ion affinity at these sites, implying a complex mechanism for how the opening degree of selectivity filter regulates ion conduction rate.

Supplementary Note 5 - Sampling.

We observe a large uncertainty of ion currents in some membranes, e.g., ion currents of the simulation replicates in POPC range from 0.2 to 13 pA (Supplementary Fig. 8). However, in other membranes, such as DEPC, it is easier to reach convergence as the protein conformational space is well confined to a specific region. This observation mainly reflects a change of the energy barrier for the conformational transition of the channel by certain membrane properties. A large uncertainty (such as in POPC) may suggest that the simulation timescale (μs for a single replica) is sufficient to sample a conformational transition but not to fully equilibrate. In this

regard, a considerable number of simulation replicates is essential to attain statistical significance. Actually, we observe that conformational changes of the two helices happen at a time scale of hundreds of nanoseconds and thus, the effects of the initial conformation propagate long in simulations. Also, ion permeation is governed by several conformational changes that correlated with each other, as described above (e.g., kinked or bent state of the inner helix, positions and orientations of Ile84 and Phe87 side chains, conformation of the outer helix). Sampling all of these elements sufficiently is challenging. Finally, we are sampling subtle conformational changes of the selectivity filter. Thus, the residue side chain flexibility, as well as the thermal fluctuation of the structure, leads to substantial fluctuations. For example, the difference in the average distances of Thr59 Oγ-Oγ between the most extreme systems we simulated is only ~0.04 nm, whereas the values distribute in the range of ~0.45-0.65 nm (Supplementary Fig. 9, i.e., a range of 0.2 nm).

Supplementary Note 6 - Simulations using the AMBER14 force field.

MthK was simulated in 8 membranes using the AMBER14 force field for the protein and the Slipids force field for the lipids, including (a) lipids with two saturated tails (DLPC, DMPC, DPPC); (b) lipids with two unsaturated tails (DOPC); (c) hybrid lipids (POPC); and (d) cholesterol containing membranes (33% molar ratio) (DLPC:cholesterol, POPC:cholesterol, and sphingomyelin:cholesterol mixtures), see Supplementary Table 7.

Conformational changes of the two transmembrane helices observed in CHARMM36m simulations are confirmed by AMBER14 simulations. AMBER14 simulations also present two states for the inner helix: a kinked state with a kink and larger bending angle, and a bent state with a smaller bending angle (Supplementary Fig. 10A). We find that the membrane properties identified to impact the bent state fraction (and ion current) in CHARMM36m simulations are also observed in AMBER14 simulations: larger membrane thickness (compare DLPC to DMPC, also compare POPC:cholesterol mixture to SSM:cholesterol mixture), cholesterol content (compare mixtures with cholesterol to membranes with saturated lipids), and larger unsaturation degree of lipid tails (compare DOPC to POPC) shift the conformational equilibrium to the kinked state (see Supplementary Fig. 10B, Supplementary Table 7). Larger membrane thickness induces smaller Pro19 Cα-Cα distance of the outer helix (clockwise rotation of the outer helix,

Supplementary Fig. 10C). The Pro19 Cα-Cα distance as a function of the bent state fraction implied partial coupling between the conformations of these two transmembrane helices (Supplementary Fig. 10D).

As in CHARMM36m simulations, transition from the kinked state to the bent state induces larger opening degree of the selectivity filter (as determined by the Thr59 Oγ-Oγ distance, Supplementary Fig. 11A). Conformation of the inner helix and the opening degree of the selectivity filter are coupled by the Ile84-Thr59 side chain interactions. As shown in Supplementary Fig. 11C-D, the bent state induces larger Ile84-Thr59 side chain distances (Supplementary Fig. 11C), which in turn pull Thr59 residues of different subunits away from each other, so that the Thr59 Oγ-Oγ distance is increased in simulations with higher bent state fraction (Supplementary Fig. 11D). The relevance of the opening degree of the selectivity filter is proved by the correlation between the ion current and the Thr59 Oγ-Oγ distance (Supplementary Fig. 11B). However, note that the POPC simulation is an exception, as the central cavity is partially dehydrated by the Phe87 side chains due to its high bent state fraction (see Supplementary Fig. 12 and Supplementary Table 7). Selectivity filter opening involves coupled motions of all the selectivity filter residues, not only the intracellular entrance (i.e., the Thr59 Oγ-Oγ distance), as suggested by the correlation between the Thr59 Oγ-Oγ distance and the backbone oxygen atom distances of Thr59 (top panel) and Gly61 (bottom panel) residues (Supplementary Fig. 11E). Note that at a given bent state fraction, the opening degree of the selectivity filter is larger in AMBER14 force fields than in CHARMM36m force fields: for instance, when the bent state fraction is ~10%, the Thr59 Oγ-Oγ distances are ~0.505 and ~0.518 nm in CHARMM36m and AMBER14 simulations, respectively (Supplementary Table 2, 6).

Two different orientations of the Phe87 side chain and the coupling between Phe87 side chain orientation and the conformation of inner helix are also found in AMBER14 simulations (Fig. 11). Note that in AMBER14 simulations using POPC membrane, the bent state fraction is ~50%, and the dehydration of the central cavity is an additional energy barrier for ion permeation, as indicated by the comparable ion current, but larger Thr59 Oγ-Oγ distance in POPC than in DMPC (Supplementary Fig. 11B).

In summary, the effects of membrane properties (thickness, unsaturation degree of lipid tails, cholesterol content) on the conformations of outer and inner helices observed in CHARMM36m simulations, as well as the correlation between the conformation of the inner helix and the structure of the permeation pathway (i.e., opening degree of the selectivity filter mediated by Ile84-Thr59 sidechain interactions, and the dehydration degree of the central cavity mediated by the sidechain orientation of Phe87), are confirmed (see supporting information for details, Supplementary Fig. 10-12, Supplementary Table 7). However, the AMBER14 simulations show a larger degree of selectivity filter opening than the CHARMM36m simulations at given bent state fraction of the inner helix. This difference might reflect slight differences of the tertiary structures of the protein in AMBER14 and CHARMM36m force fields. Ion permeation rates only show minor differences between simulations using the two force fields at a given Thr59 Oγ-Oγ distance, suggesting ion-protein interactions are comparable for these two force fields.

Supplementary Note 7 - Effects of membrane thickness and cholesterol concentration on ion permeation rate revealed by experiments in literature.

The effects of membrane thickness on the open probability and ion permeation rate of potassium channels are also observed by experiments. Callahan et al.[24] revealed that KcsA is more active in thin bilayers (14:1 to 18:1 PC lipids) than in thick bilayers (20:1 and 22:1 PC lipids) and ascribed the different activities to the increased energy barrier for conformational changes between the open and closed states in thicker membranes, in line with our simulations of MthK. Yuan et al.[23] investigated the effects of membrane thickness on ion conduction of the large-conductance Ca^{2+}-activated potassium channel (BK_{Ca}), a homologue of MthK. An optimum conductance was observed in a 18:1 lipids (corresponding to DOPC), while both thinner (14:1, thinner than DYPC) and thicker (22:1 and 24:1, corresponding to DEPC and DNPC) membranes reduced the ion permeation rate, indicating the same trend as revealed in our simulations of MthK (Fig. 2). In another work from the same group,[22] a slightly different result was found: ion conductance decreased with increasing lipid tail length from 14:1 to 22:1, and then increased with further increase of the tail length to 24:1 or sphingomyelin. This result is partially in line with our simulations using DOPC and DEPC lipids. Increased conductance with further increase of membrane thickness was explained in the study by the interplay between the transmembrane domain and the intracellular domain in the presence of a dramatical thickness

mismatch. However, we used only the pore domain of the channel in simulations and hence cannot test their hypothesis further. They also showed modified stability of both open and closed states and an increased energy barrier of conformational transition between them in thicker membranes, which may fit with the changes of the open probability of MthK we describe in our proposed model. Chang et al. and Crowley et al.'s experiments,[25,26] indicated decreased ion permeation rate and open probability of BK_{Ca} as a result of increased cholesterol concentration, which highlight the role of lateral pressure and is consistent with our simulations of cholesterol containing membranes.

In the case of MthK in different bilayers in our simulations, thicker membranes are more ordered at given temperature due to their higher phase transition temperature, which may increase the lateral stress. Cholesterol also results in a larger lateral pressure in the bilayer by inducing more ordered packing of the phospholipids. We propose that, the larger lateral pressure may push the outer helices and induce their clockwise rotation (Fig. 3F, Fig. 4A), so that the kinked state of the inner helices is stabilized (Fig. 3G). However, we do not rule out the possibility that the larger lateral pressure affects the conformation of the inner helix directly.

Supplementary Methods.

Setups of simulations with restraints on the protein conformation.

We conducted a series of simulations of the MthK potassium channel with restraints on the inner and outer helices to further evaluate effects of the conformational changes revealed by unrestrained simulations on the ion permeation rate.

Simulations with restraints on the inner helices. Two states were found for the inner helix: the kinked state which has a kink in the vicinity of Gly83, and the bent state which does not have this kink. To control the conformation of the inner helix in the kinked and bent state, we applied harmonic restraints of four backbone "hydrogen bonds": Ile80O-Ile84HN, Val81O-Gly85HN, Leu82O-Thr86HN, and Gly83O-Phe87HN (Supplementary Table. 1). O refers to the carbonyl oxygen, while HN refers to hydrogen atom of the amide group. Target distances and force constants for the kinked and bent states are shown in Supplementary Table 1. These simulations were conducted for the POPC membranes to restrain all four inner helices at the kinked state,

and for the DOPC membranes to restrain all four or one of the four (while leaving the other three subunits free) inner helices at the bent state, respectively (see Supplementary Table 4).

Phe87 is a residue close to the kink region of the inner helix. It was observed to reside in the central cavity in the bent state of the inner helix and dehydrate the cavity. When all four subunits were restrained at the bent state, a partially unwinding was observed for the helical turn immediately following the kink (i.e., between residues Gly85 and Ala90) in some simulations, probably due to steric hindrance when all four Phe87 side chains resided in the central cavity. This partial unwinding allowed Phe87 side chains to reside out of the central cavity. We therefore performed simulations with harmonic restraints of the backbone "hydrogen bonds" of this region including Ile84O-Ala88HN, Gly85O-Val89HN, Thr86O-Ala90HN, and Phe87O-Val91HN, in addition to those restrained in the bent state. These addition restraints may stabilize the Phe87 side chain conformation in the central cavity. This simulation was performed in DOPC membranes to test the ion permeation when the central cavity is completely occluded and dehydrated. It is referred as occluded state simulation, see Supplementary Table 1 for the target values and force constants of distance restraints.

Simulations with restraints on the outer helices. We performed simulations in which the Pro19 Cα-Cα distance was restrained, in order to test effects of the conformation of the outer helix on ion conduction. Both the distances between atoms of adjacent subunits and the distances between atoms of opposite subunits were controlled by the pulling code of GROMACS package using a force constant of 500 kJ/(mol nm^2). This simulation was performed for the POPC system (in which the ion permeation reached an optimum) with a target value that was found in simulations with blocked ion conduction, as well as the DEPC system (in which ion permeation was inhibited) with a target value that found in simulations with optimum ion current, see Supplementary Table 4.

Simulations with restraints on the Ile84 side chain distances. Ile84 is a residue at the inner helix and its side chain has interactions with Thr59, the first residue constituting the selectivity filter. In order to simulate the protein with different degrees of opening of the selectivity filter with least effects on the flexibility of the selectivity filter conformation, we restrained the

distances between the centres of mass (COM) of the Ile84 side chains to a series of values using the above protocol for restraining the Pro19 Cα-Cα distance. In this case, a force constant of 5000 $kJ \cdot mol^{-1} \cdot nm^{-2}$) was used. The target distances reported in Supplementary Table 5 are the distances between opposite residues. This simulation was conducted for MthK in the DLPC and POPC membranes, see Supplementary Table 5 and 6.

Parameters of molecular dynamics simulations.

Molecular dynamics simulations were conducted an integration time step of 2 fs. The CHARMM36m force field[27] was used for proteins and standard CHARMM36 parameters was used for lipids[28], the TIP3P water model[29], and ions. All hydrogen containing bonds were constrained by the LINCS algorithm.[30] The pressure was maintained at 1 bar using the semi-isotropic Parrinello-Rahman barostat[31,32] and a relaxation time of 5 ps. The Nose-Hoover thermostat[33,34] was applied to maintain the temperature at 323 K with a relaxation time of 1 ps. We used the force switch method[35] to turn off the van der Waals interactions from 1.0 to 1.2 nm. Long range electrostatic interactions were computed with the Particle Mesh Ewald[36,37] method, using a real space cut-off value of 1.2 nm, a Fourier grid spacing of 0.12 nm, and an interpolation order of 4 for the Ewald mesh. For the simulations using AMBER14 force field for the protein and Slipids force filed for the lipids, the same parameters were used, except for a cut-off value of 1.0 nm for van der Waals and long range electrostatic interactions and a v-rescale thermostat[38]. Each system contained 1 MthK protein, ~140-180 lipids, ~10,000 water molecules, and ~350-400 potassium and chloride ions depending on membrane compositions.

The systems were first equilibrated in six steps with gradually removed restraints on the protein and lipids, using the default scheme suggested for GROMACS5.1 by CHARMM-GUI. Three parallel equilibrium simulations without restraints and transmembrane voltage were then performed for 0.1-0.3 μs for each system. At last, 2-4 snapshots randomly selected from the equilibrium simulations were used as initial states of the production simulations in which a transmembrane voltage was applied.

Our simulations were performed at 323 K to investigate lipid-protein interactions in a liquid phase state environment (either Lα or liquid ordered), as some of the membranes, e.g. DPPC,

may form a gel phase at lower temperatures. This higher temperature also accelerated ion permeation rate and allowed us to observe more ion permeation events at given simulation time. In addition, this temperature may accelerate the conformational transition between different states of the protein in our simulations. Considering the energy barrier the protein needs to overcome during the conformational transition, the chosen enhanced temperature aids sampling transition events while remaining in the physiological range for MthK a potassium channel from the thermophile *Methanothermobacter thermautotrophicus*.

Data analysis.

To evaluate the ion conduction rate, we used the FORTRAN code in Kopec et al's work[39] to count the number of permeation events. The ion current across the channel was calculated by dividing the permeated charges by simulation time.

The distances between Ile84 and Thr59 side chains were calculated for the four subunits separately and the averages are reported. We also calculated the Ile84-Thr59 distance for the subunits in the kinked and bent states separately, and compared the corresponding averages. Phe87 side chain had two conformations in our simulations: in one conformation, its side chain interacted with hydrophobic residues from the adjacent subunit, while in the other conformation, its side chain pointed toward the central cavity (Fig. 5). In order to characterize the orientation of Phe87 side chain, we defined the center of mass (COM) of the four Phe87 Cα atoms as the center of the Phe residues (CoF), and calculated the distances between the COM of Phe87 side chain and the CoF for the four subunits separately, and reported the averages. A smaller average value for the Phe87-CoF distance is expected if more Phe87 side chains point toward the central cavity. The other distances reported in this work are the average distances between the corresponding residues/atoms from opposite subunits.

A cutoff value of 0.34 nm was used for the Val81O-Gly85HN distance to discriminate the kinked and bent states in the simulations: it was considered as in the kinked state if the Val81O-Gly85HN distance is larger than 0.34 nm. Two vectors are fitted by singular value decomposition of the backbone atom coordinates of res. 71-81 and res. 85-99 respectively. The bending angle of the inner helix was defined as the angle between these two vectors.

Functional mode analysis (FMA) attempts to correlate a functional quantity that describes the function of a protein, to the collective motions of the protein. In this work, we choose the bending angle of the inner helix of one of the four subunits as the functional quantity and performed FMA of the tetramer to evaluate the protein conformational changes relevant to the bending of this inner helix. This was done for concatenated trajectories of simulations in POPC membranes, as well as concatenated simulation trajectories of different systems, and the same conclusions were obtained.

The pore radius was calculated by HOLE[40] for each frame of the trajectories and the average was reported. To calculate the density of potassium and water along the permeation pathway, we superimposed the systems based on the selectivity filter (specifically, the heavy atoms of Thr59 and the backbones of Val60, Gly61 and Tyr62) and counted the potassium/water molecules located within a cylinder whose axis is parallel to the membrane normal and passes through the center of mass of the Cα atoms of Glu96, a residue at the entrance of the central cavity. We used a radius of 1.2 nm for the cylinder, approximately the size of the entrance of the central cavity. The density profile was calculated as the number distribution and normalized to the value in bulk. The “free energy” profile was generated based on the density profile.

Supplementary Table 1. Distance restraints to control the conformation of the inner helix.

Distance Restrained	Target value in kinked state (nm)	Target value in bent state (nm)	Target value in occluded state (nm)	Force constant ($kJ \cdot mol^{-1} \cdot nm^{-2}$))
**Ile80O-Ile84HN**	0.27	0.20	0.20	3500
**Val81O-Gly85HN**	0.43	0.24	0.24	3500
**Leu82O-Thr86HN**	0.35	0.21	0.21	3500
**Gly83O-Phe87HN**	0.20	0.20	0.20	1500
**Ile84O-Ala88HN**	-	-	0.22	3500
**Gly85O-Val89HN**	-	-	0.25	3500
**Thr86O-Ala90HN**	-	-	0.21	3500
**Phe87O-Val91HN**	-	-	0.21	3500

Supplementary Table 2. Simulations of MthK in different membranes using CHARMM36m force field. Data are presented as mean values ± SEM. Source data are available as a Source Datafile.

Membrane	Thickness (nm)	Sim. Time (μs × No.)	Curr. (pA)	Distance (nm)				Bent state fraction
				Thr59 Oγ-Oγ	Pro19 Cα-Cα	Ile84-Thr59 Sidechain COM	Phe87-CoF	
**Lipids with two saturated tails**								
DLPC, 12:0/12:0	3.15±0.01	1.5×40	4.15±0.35	0.507±0.001	3.54±0.01	0.547±0.001	1.039±0.006	12%±1%
DMPC, 14:0/14:0	3.63±0.01	0.5×30	6.19±0.61	0.515±0.001	3.56±0.02	0.560±0.003	0.919±0.014	32%±3%
DPPC, 16:0, 16:0	4.19±0.01	0.5×40	3.81±0.40	0.506±0.001	3.36±0.01	0.545±0.001	1.048±0.007	8%±2%
**Lipids with two unsaturated tails**								
DYPC, 16:1(n-9)/16:1(n-9)	3.56±0.01	0.5×20	3.54±0.66	0.505±0.001	3.46±0.03	0.548±0.002	1.008±0.015	15%±3%
DOPC, 18:1(n-9)/18:1(n-9)	3.86±0.01	0.5×40	4.61±0.40	0.507±0.001	3.37±0.03	0.549±0.002	1.042±0.008	11%±2%
DGPC, 20:1(n-11)/20:1(n-11)	4.25±0.01	0.5×25	1.15±0.20	0.499±0.001	3.21±0.01	0.539±0.001	1.072±0.002	2%±1%
DEPC, 22:1(n-13)/22:1(n-13)	4.60±0.01	0.5×20	0.92±0.11	0.497±0.001	3.17±0.01	0.536±0.001	1.068±0.001	1%±0.2%
DNPC, 24:1(n-15)/24:1(n-15)	4.90±0.01	0.5×10	0.61±0.11	0.498±0.001	3.13±0.01	0.535±0.002	1.073±0.002	1%±0.2%
**Hybrid lipids**								
POPC, 16:0/18:1(n-9)	3.92±0.01	1.5×40	6.29±0.52	0.515±0.001	3.55±0.02	0.568±0.003	0.937±0.016	34%±3%
**Cholesterol containing membranes, with cholesterol molar ratio of 33%**								
DLPC:Cholesterol	3.63±0.01	0.5×30	1.60±0.35	0.501±0.001	3.40±0.02	0.538±0.002	1.069±0.005	4%±1%
POPC:Cholesterol	4.24±0.02	0.5×10	0.90±0.21	0.499±0.001	3.27±0.03	0.534±0.002	1.052±0.011	4%±2%

Supplementary Table 3. Correlation between ion permeation and the fraction of bent state of the inner helix. Simulation replicates in DLPC, DYPC, DOPC and DPPC are divided to two groups based on their bent state fraction in the trajectories. The average currents and distances are shown for each group. Data are presented as mean values ± SEM. Source data are available as a Source Datafile.

Membrane		Sim. Time (μs×No.)	Curr. (pA)	Thr59 Oγ-Oγ (nm)	Pro19 Cα-Cα (nm)	Ile84-Thr59 COM (nm)	Phe87-CoF (nm)	Bent state fraction
**DLPC**	**group1**	1.5×15	6.19±0.38	0.513±0.001	3.58±0.01	0.555±0.002	1.002±0.011	22%±1%
	**group2**	1.5×25	2.93±0.31	0.504±0.001	3.51±0.02	0.542±0.001	1.061±0.003	7%±1%
**DYPC**	**group1**	0.5×11	5.15±0.90	0.510±0.002	3.54±0.03	0.555±0.002	0.958±0.015	25%±2%
	**group2**	0.5×9	1.57±0.39	0.501±0.001	3.35±0.03	0.538±0.002	1.069±0.006	3%±1%
**DOPC**	**group1**	0.5×17	5.96±0.47	0.512±0.001	3.45±0.02	0.556±0.002	1.012±0.015	22%±2%
	**group2**	0.5×23	3.62±0.52	0.503±0.001	3.31±0.01	0.542±0.001	1.064±0.002	4%±1%
**DPPC**	**group1**	0.5×10	6.35±0.93	0.513±0.002	3.45±0.02	0.554±0.004	0.995±0.016	23%±4%
	**group2**	0.5×30	2.97±0.32	0.504±0.001	3.33±0.01	0.543±0.001	1.066±0.003	4%±1%

Supplementary Table 4. Restraining the conformation of the two transmembrane helices modulates ion currents. Data are presented as mean values ± SEM. Source data are available as a Source Datafile.

Membrane	Rest. Target Val.	Sim. Time (μs×No.)	Curr. (pA)	Thr59 Oγ-Oγ (nm)	Pro19 Cα-Cα (nm)	Ile84-Thr59 COM (nm)	Phe87-CoF (nm)	Bent state fraction
**Restrain Pro19 Cα-Cα distance**								
**POPC**	3.20 nm	0.5×10	1.99±0.38	0.502±0.001	3.21±0.01	0.540±0.002	1.063±0.002	24%±2%
**DEPC**	3.55 nm	0.5×10	4.73±1.11	0.509±0.002	3.53±0.01	0.549±0.002	1.056±0.002	8%±2%
**Restrain inner helix conformation**								
**POPC**	**Kinked state**	0.5×10	0.38±0.06	0.497±0.001	3.28±0.02	0.533±0.001	1.086±0.002	0%±0.1%
**DOPC**	**Bent state**[a] (1 subunit)	0.5×10	5.90±0.52	0.512±0.001	3.43±0.03	0.554±0.002	0.975±0.009	28%±2%
**DOPC**	**Bent state**[b] (4 subunits)	1.0×10	2.88±1.05	0.533±0.003	3.75±0.02	0.642±0.006	0.703±0.061	100%±0.2%
**DOPC**	**Bent state (F87A)** (4 subunits)	0.5×10	13.33±1.04	0.529±0.002	3.58±0.03	0.614±0.002	-	100%±0.2%
**DOPC**	**Occluded state**[c] (4 subunits)	0.5×10	0.71±0.10	0.527±0.003	3.82±0.02	0.664±0.003	0.514±0.009	100%±0.2%
**DOPC**	**Occluded state (3 subunits)**	0.5×10	1.93±0.26	0.531±0.002	3.69±0.02	0.622±0.002	0.730±0.005	76%±1%
**DOPC**	**Occluded state**[d] **(2 subunits)**	0.5×10	10.32±0.86	0.528±0.002	3.62±0.03	0.591±0.003	0.777±0.006	55%±2%
**DOPC**	**Occluded state (1 subunit)**	0.5×10	5.45±0.90	0.512±0.003	3.49±0.04	0.559±0.004	0.944±0.010	32%±3%

[a] The numbers indicated in the parentheses are the number of subunits restrained in the indicated state. The remaining subunits are left free. [b] 1 μs simulation was performed for each simulation replicate, however, data of the last 0.5 μs was reported, as the first 0.5 μs simulation did not converge due to a partial unwinding of the helical turn immediately following the kink (from Gly85 to Val89). [c] A snapshot at 500 ns in a bent state simulation was used as the initial conformation. [d] Two adjacent subunits are restrained.

Supplementary Table 5. Simulations with the Ile84 side chain distances restrained in POPC and DLPC membranes. Data are presented as mean values ± SEM. Source data are available as a Source Datafile.

Target Dist.(nm)	POPC			DLPC		
	Sim. Time (μs×No.)	Thr59 Oγ-Oγ	Curr. (pA)	Sim. Time (μs×No.)	Thr59 Oγ-Oγ	Curr. (pA)
1.62	0.5×20	0.528±0.002	16.13±1.50	0.5×10	0.525±0.002	16.43±1.81
1.60	0.5×10	0.523±0.002	10.86±1.41	0.5×10	0.517±0.002	11.09±1.25
1.57	0.5×10	0.514±0.001	7.12±1.18	0.5×10	0.508±0.001	5.83±0.81
1.55	0.5×10	0.510±0.001	5.38±0.96	0.5×10	0.505±0.001	3.24±0.71
1.52	0.5×10	0.504±0.001	3.08±0.53	0.5×10	0.501±0.001	1.57±0.45
1.50	0.5×10	0.505±0.001	3.46±0.51	0.5×10	0.498±0.001	0.74±0.19

Supplementary Table 6. Simulations with the Ile84 side chain distances restrained in POPC membrane. Target values of the restraint are 1.62 and 1.65 nm, respectively, which result in an average Thr59 Oγ-Oγ distance of ~0.528 and ~0.538 nm. Simulation replicates are divided to two groups based on a cutoff value of 12 (1.62 nm simulation) and 15 pA (1.65 nm simulation) for the current. Average currents, distances, and bent state fractions of all replicates, and the replicates in each group are shown. Data are presented as mean values ± SEM. Source data are available as a Source Datafile.

Membrane	Category	Sim. Time (μs×No.)	Curr. (pA)	Thr59 Oγ-Oγ (nm)	Pro19 Cα-Cα (nm)	Ile84-Thr59 COM (nm)	Phe87-CoF (nm)	Bent state fraction
**Restrain Ile84 side chain distance at 1.62 nm, current cutoff value is 10 pA**								
**POPC**	All	0.5×20	16.13±1.50	0.528±0.001	3.59±0.01	0.578±0.003	0.852±0.021	44%±3%
**POPC**	Smaller Curr.	0.5×6	8.38±0.66	0.529±0.002	3.59±0.02	0.585±0.006	0.762±0.034	57%±4%
**POPC**	Larger Curr.	0.5×14	19.46±1.33	0.527±0.001	3.60±0.02	0.576±0.003	0.891±0.018	39%±3%
**Restrain Ile84 side chain distance at 1.65 nm, current cutoff value is 15 pA**								
**POPC**	All	0.5×20	13.40±2.16	0.538±0.001	3.63±0.01	0.611±0.004	0.725±0.021	65%±3%
**POPC**	Smaller Curr.	0.5×13	6.93±0.86	0.537±0.001	3.62±0.02	0.621±0.003	0.666±0.013	71%±2%
**POPC**	Largeer Curr.	0.5×7	25.41±1.60	0.541±0.001	3.65±0.03	0.594±0.004	0.836±0.020	54%±5%

Supplementary Table 7. Simulations of MthK in different membranes using AMBER14 force field for the protein and the Slipids force field for the lipids. Data are presented as mean values ± SEM. Source data are available as a Source Datafile.

Membrane	Thickness (nm)	Sim. Time (μs × No.)	Curr. (pA)	Distance (nm)				Bent state fraction
				Thr59 Oγ-Oγ	Pro19 Cα-Cα	Ile84-Thr59 Sidechain COM	Phe87-CoF	
**Lipids with two saturated tails**								
DLPC, 12:0/12:0	3.04±0.01	0.5×20	11.17±0.66	0.518±0.001	3.64±0.01	0.526±0.001	1.054±0.006	10%±2%
DMPC, 14:0/14:0	3.42±0.02	0.5×10	14.68±0.69	0.526±0.002	3.64±0.02	0.541±0.004	0.965±0.020	28%±5%
DPPC, 16:0, 16:0	3.81±0.02	0.5×10	13.94±1.06	0.524±0.003	3.49±0.02	0.532±0.003	1.019±0.013	19%±4%
**Lipids with two unsaturated tails**								
DOPC, 18:1(n-9)/18:1(n-9)	3.65±0.02	0.5×10	11.60±0.62	0.523±0.003	3.56±0.02	0.532±0.004	1.038±0.016	20%±5%
**Hybrid lipids**								
POPC, 16:0/18:1(n-9)	3.67±0.02	0.5×20	13.67±0.57	0.532±0.002	3.60±0.01	0.567±0.005	0.880±0.015	51%±3%
**Cholesterol containing membranes, with cholesterol molar ratio of 33%**								
DLPC:Cholesterol	3.49±0.01	0.5×10	7.91±1.04	0.513±0.002	3.53±0.01	0.525±0.002	1.049±0.004	7%±2%
POPC:Cholesterol	4.02±0.02	0.5×10	8.04±0.98	0.513±0.001	3.45±0.03	0.523±0.003	1.010±0.021	10%±3%
SSM:Cholesterol 18:1(n-4)/18:0	4.43±0.01	0.5×10	5.19±0.56	0.505±0.001	3.26±0.02	0.513±0.001	1.050±0.002	2%±1%

Supplementary Figure 1. Coupling between the conformations of the two transmembrane helices. Left panel: Simulations in DLPC, DYPC, DOPC, and DPPC membranes. Simulation replicates are divided in two groups (labelled as gp1 and gp2, respectively), and the average distances and bent state fractions are calculated separately for the two groups; right panel: Simulations with restraints of the inner and outer helices (open symbols). Simulations in corresponding membranes without restraints are also shown for comparison (filled symbols). Number of replicates for each data point in the left panel is the same with Fig. 3D. Each data point of the restrained simulations in the right panel includes 10 independent simulations. Data are presented as mean values ± SEM. Source data are available as a Source Datafile.

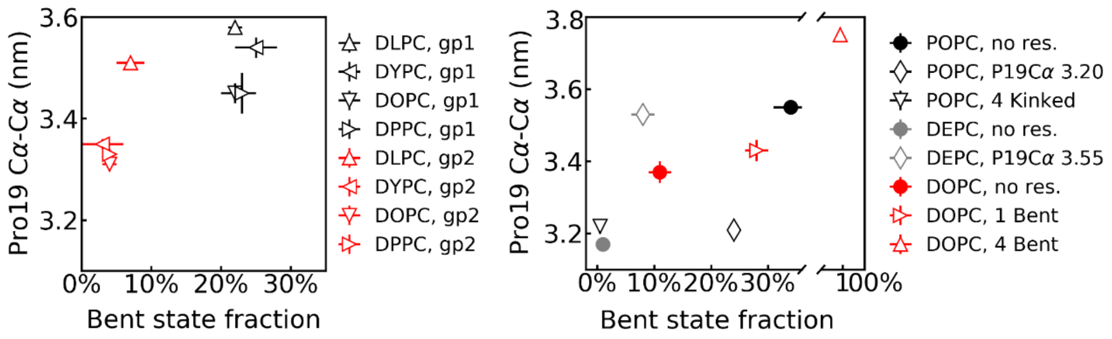

Supplementary Figure 2. Restraining the outer helix induces changes of the bending angle of the inner helix. Results are compared for the simulations with and without restraints of the Pro19 Cα-Cα distance. In POPC, Pro19 Cα-Cα distance is restrained to a smaller value (compared with the simulations without restraints), while it is restrained to a larger value in DEPC. Source data are available as a Source Datafile.

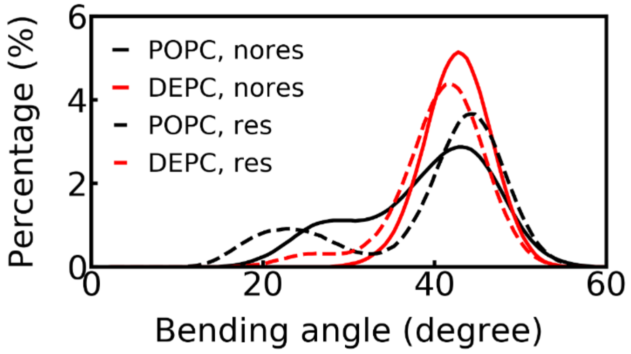

Supplementary Figure 3. Ion current as a function of the Thr59 Oγ-Oγ distance. (A) Simulations using different membranes. (B) Simulations in DLPC, DYPC, DOPC, and DPPC membranes. Simulation replicates are divided in two groups (labelled as gp1 and gp2, respectively), and the average currents and distances are calculated separately for the two groups. (C) Simulations with restraints of the inner and outer helices (open symbols). Simulations in corresponding membranes without restraints are also shown for comparison (filled symbols). Number of replicates for each data point in panels A, B and C is the same with Fig. 2, Fig. 3D, and the right panel of Supplementary Fig. 1, respectively. Data are presented as mean values ± SEM. Source data are available as a Source Datafile.

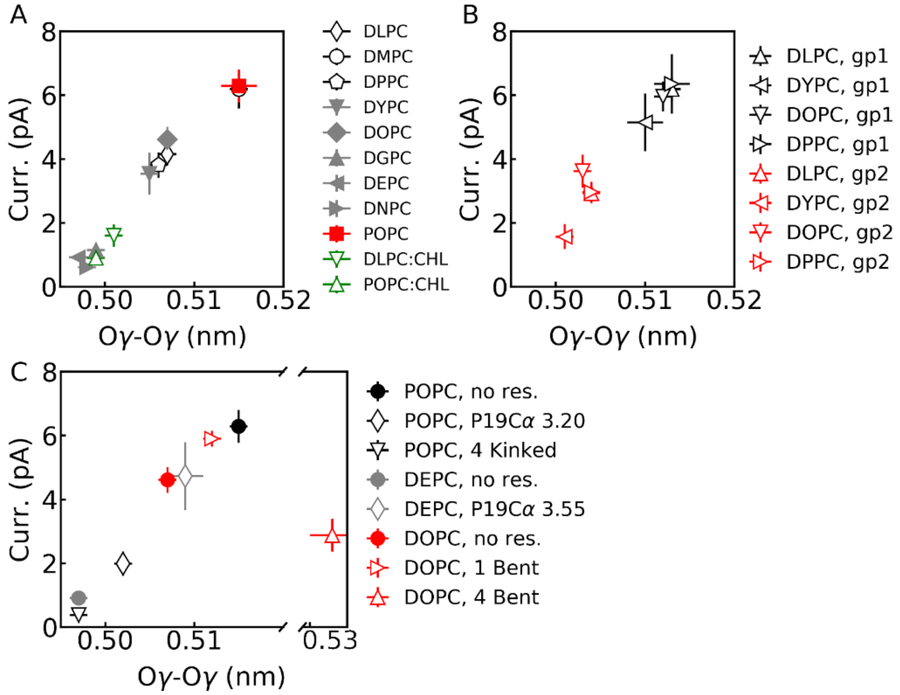

Supplementary Figure 4. Correlation between the Thr59 Oγ-Oγ distance and the Ile84-Thr59 side chain distance. Left panel: Simulations in DLPC, DYPC, DOPC, and DPPC membranes. Simulation replicates are divided in two groups (labelled as gp1 and gp2, respectively), and the average distances are calculated separately for the two groups; right panel: Simulations with restraints of the inner and outer helices (open symbols). Simulations in corresponding membranes without restraints are also shown for comparison (filled symbols). Number of simulation replicates for each data point in the left and right panels is the same with Fig. 3D and the right panel of Supplementary Fig. 1, respectively. Data are presented as mean values ± SEM. Source data are available as a Source Datafile.

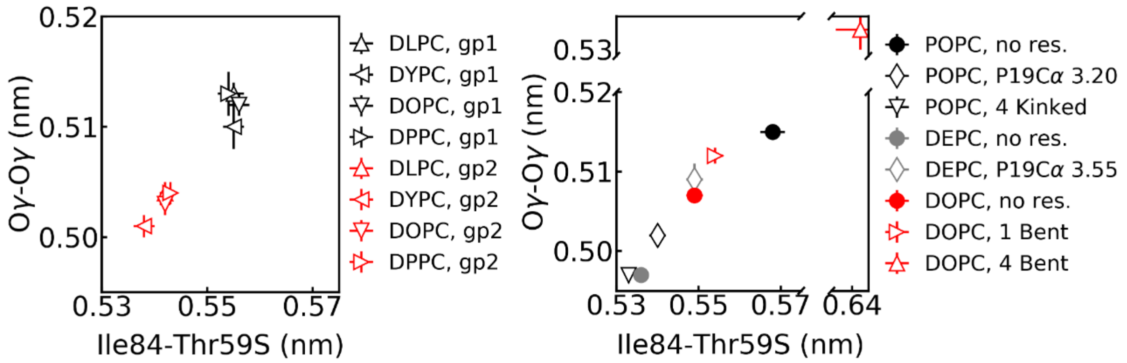

Supplementary Figure 5. Correlation between the potassium occupancy at the selectivity filter and the protein conformation. Potassium occupancies at S4 and S3 binding sites as a function of the Thr59 Oγ-Oγ distance (top panel), and the Ile84-Thr59 sidechain distance (bottom panel). Number of simulation replicates for each data point is the same with Fig. 2. Data are presented as mean values ± SEM. Source data are available as a Source Datafile.

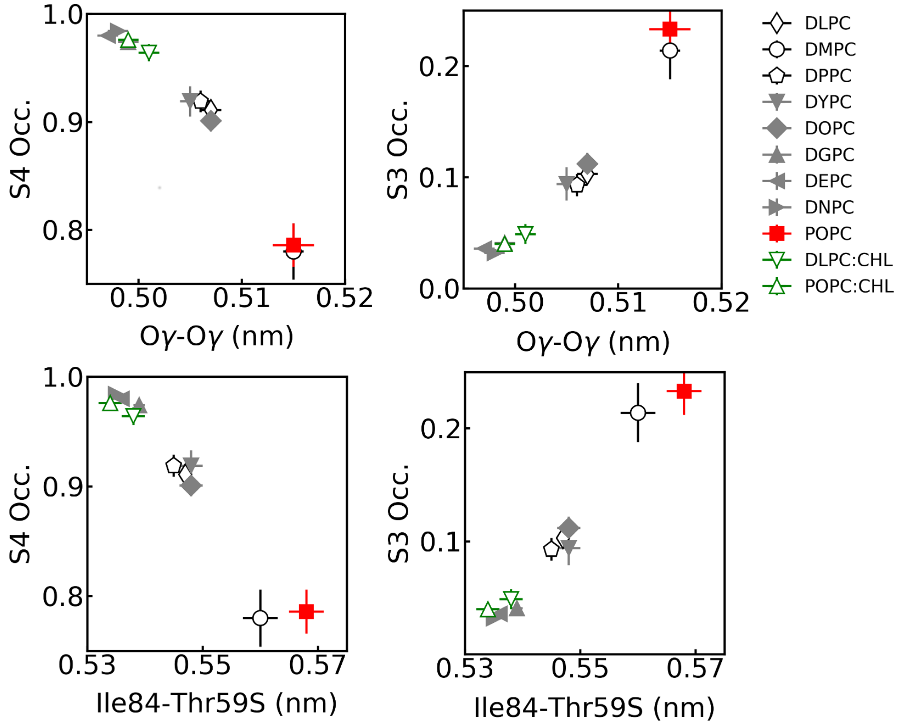

Supplementary Figure 6. Correlation between Phe87-CoF distance and the bent state fraction. Left panel: Simulations in DLPC, DYPC, DOPC, and DPPC membranes. Simulation replicates are divided in two groups (labelled as gp1 and gp2, respectively), and the average distances and bent state fractions are calculated separately for the two groups; right panel: Simulations with restraints of the inner and outer helices (open symbols). Simulations in corresponding membranes without restraints are also shown for comparison (filled symbols). Number of simulation replicates for each data point in the left and right panels is the same with Fig. 3D, and the right panel of Supplementary Fig. 1, respectively. Data are presented as mean values ± SEM. Source data are available as a Source Datafile.

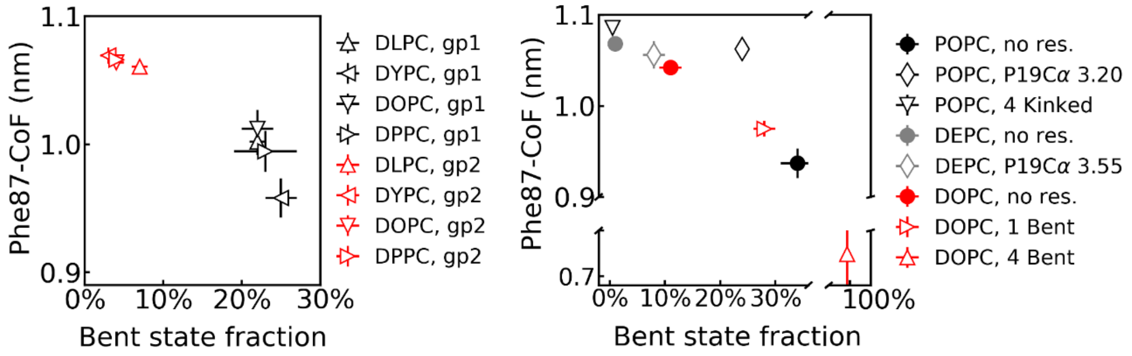

Supplementary Figure 7. Dehydration of the central cavity reduce ion conduction rate. Simulations in which the Ile84 side chain distance is restrained at 1.62 and 1.65 nm are analyzed. The simulations are divided in two categories based on cutoff values of 12 pA and 15 pA for the currents, respectively. The (A) pore radius, "free energy" profiles of water and potassium, as well as the (B) average currents are shown for each category. "Energy barriers" of potassium are indicated in the text label of panel B. Ion currents of the simulation replicates in panel B are shown as black dots. The numbers of replicates are indicated above the X axis. Data are presented as mean values ± SEM. Source data are available as a Source Datafile.

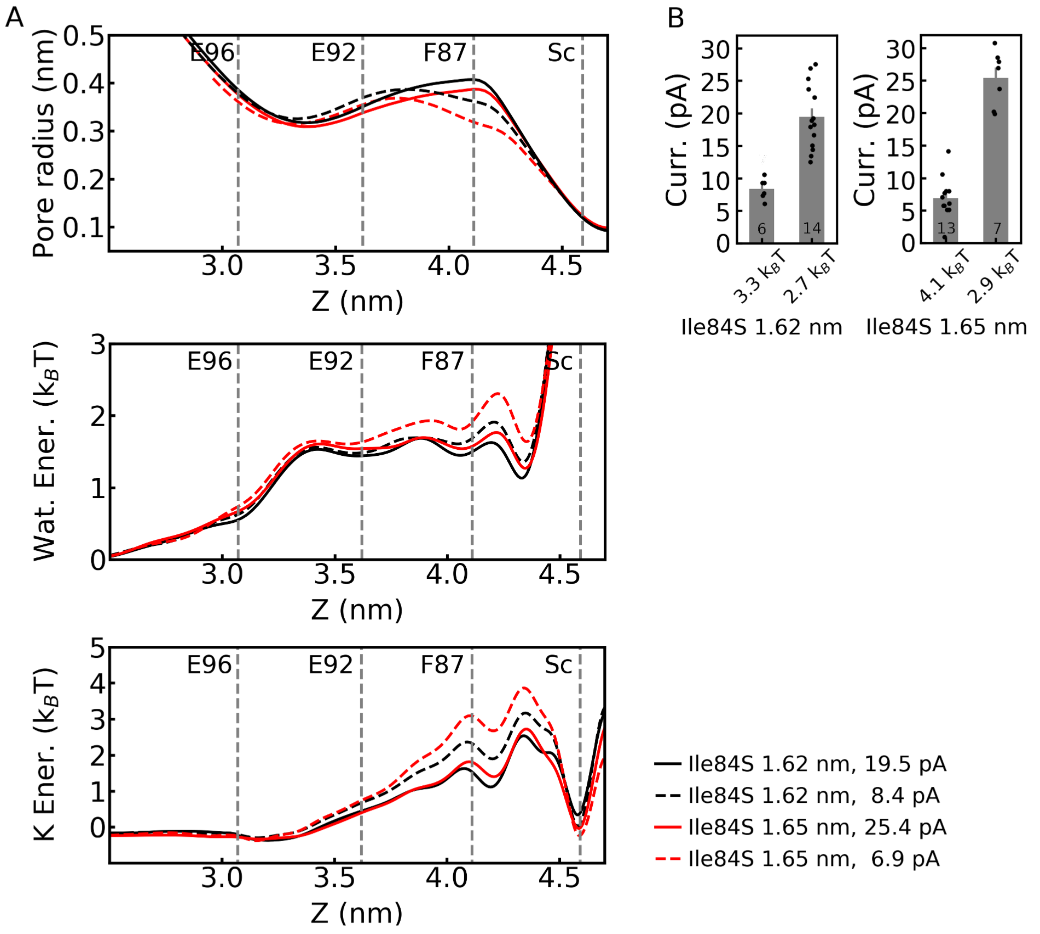

Supplementary Figure 8. Ion current as a function of the Thr59 Oγ-Oγ distance for simulation replicates in POPC. A large uncertainty is observed for ion permeation rate. Source data are available as a Source Datafile.

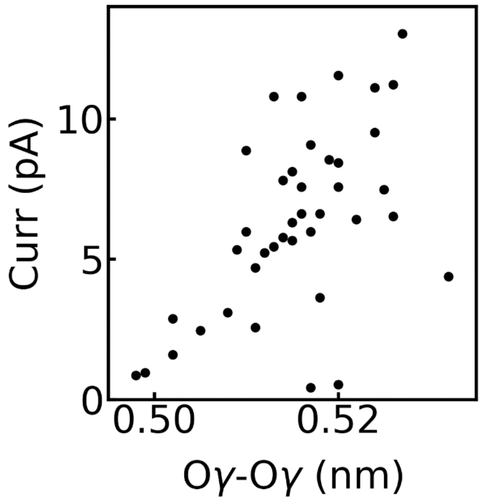

Supplementary Figure 9. Distributions of Thr59 Oγ-Oγ distances. Simulations in DLPC, DMPC and DEPC are shown as examples. Source data are available as a Source Datafile.

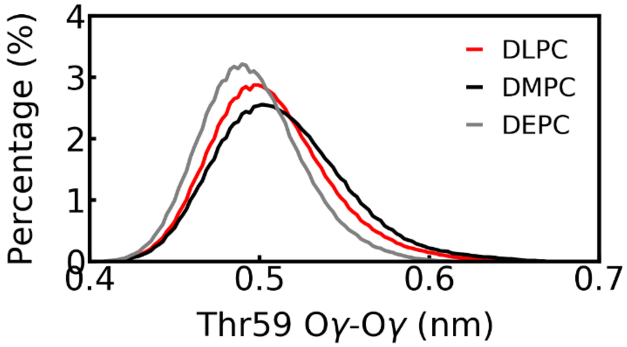

Supplementary Figure 10. Conformational changes of the two transmembrane helices in AMBER14 simulations. (A) Histogram as a function of Val81O-Gly85HN distance and the bending angle of the inner helix. (B) Bent state fraction of the inner helix as a function of the bilayer thickness. (C) Coupling of the conformations of the inner (characterized by the bent state fraction) and outer (characterized by the Pro19 Cα-Cα distance) helices. (D) Pro19 Cα-Cα distance as a function of membrane thickness. As in CHARMM36m simulations, the simulations are conducted in membranes composed of saturated lipids (DLPC, n=20; DMPC, n=10; DPPC, n=10;), unsaturated lipids (DOPC, n=10), and hybrid lipids (POPC, n=20), and in cholesterol-containing membranes (DLPC:CHOL, n=10; POPC:CHOL, n=10; SSM:CHOL, n=10). n indicates the number of simulation replicates. Data are presented as mean values ± SEM. Source data are available as a Source Datafile.

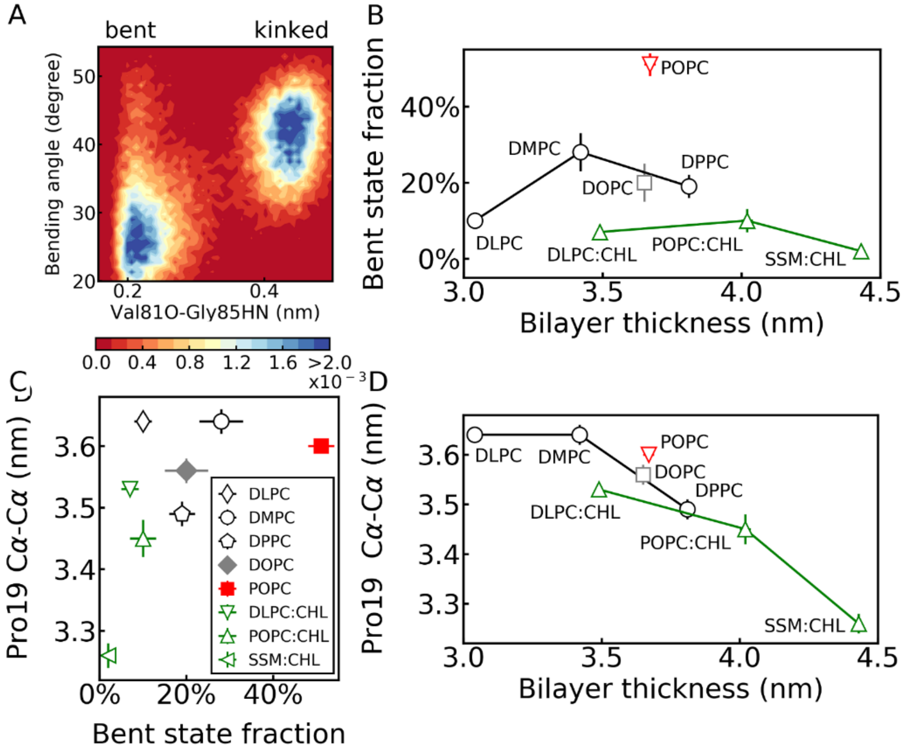

Supplementary Figure 11. Gating of the selectivity filter by the kink of the inner helix in AMBER14 simulations. (A) Thr59 Oγ-Oγ distance as a function of the bent state fraction of the protein. (B) Ion current as a function of Thr59 Oγ-Oγ distance. (C) Histogram as a function of the Ile84-Thr59 side chain distance and the Val81O-Gly85HN distance. Subunits in the kinked state show smaller Ile84-Thr59 distance. (D) The Thr59 Oγ-Oγ distance as a function of the Ile84-Thr59 side chain distance. (E) Correlation between the Thr59 Oγ-Oγ distance and the backbone oxygen atom distances of Thr59 (top) and Gly61 (bottom). Panels A, B, D and E share the same legend. Number of independent replicates for each data point is the same with Supplementary Fig. 10. Data are presented as mean values ± SEM. Source data are available as a Source Datafile.

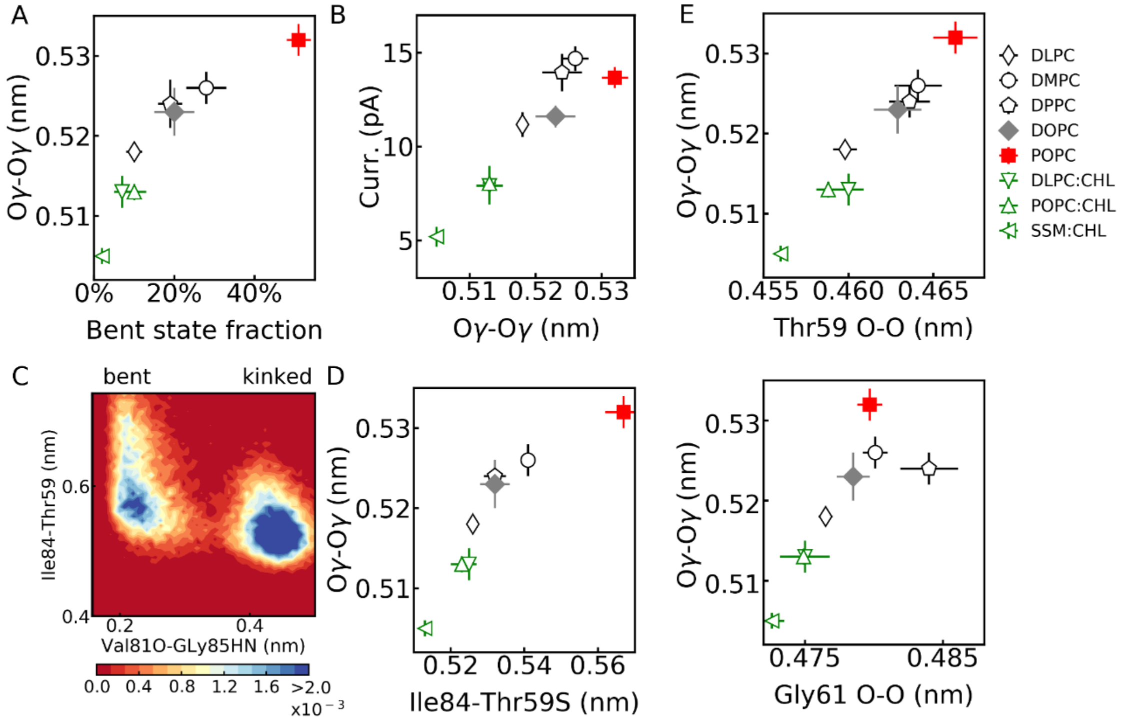

Supplementary Figure 12. Gating of the central cavity by the kink of the inner helix in AMBER14 simulations. Left panel: histogram as a function of the Val81O-Gly85HN distance (as a measure of the inner helical conformation) and the Phe87-CoF distance (as a measure of the Phe87 side chain orientation). Result of the simulations in POPC is show as an example; right panel: the Phe87-CoF distance as a function of the bent state fraction of the inner helix. Number of independent replicates for each data point in the right panel is the same with Supplementary Fig. 10. Data are presented as mean values ± SEM. Source data are available as a Source Datafile.

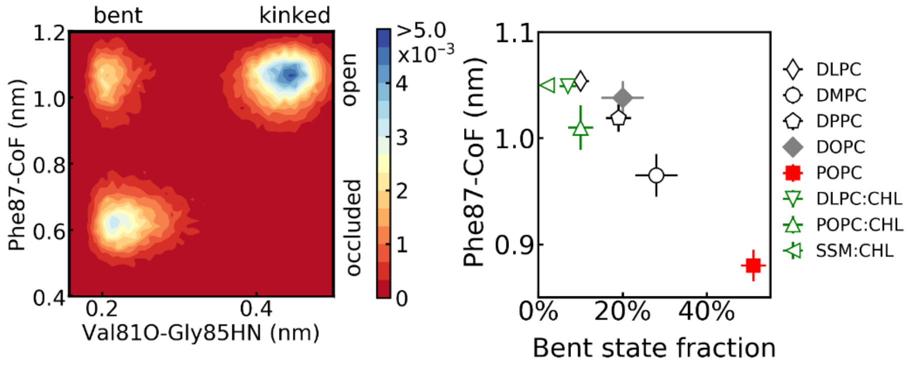

Supplementary Figure 13. Pro19 residue is buried more deeply in the hydrophobic region in thicker membranes. Normalized distributions of the density of Pro19 Cα atoms and the phosphorous atoms of lipids along the membrane normal are compared for the DOPC and DGPC simulations. Pay attention to the relative position of Pro19 and lipid headgroups.

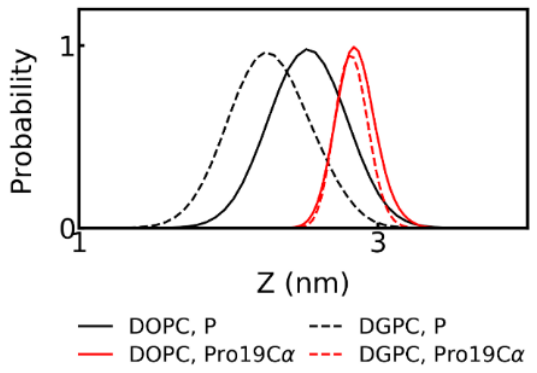

Supplementary References.

1. Tucker, S. J. & Baukrowitz, T. How highly charged anionic lipids bind and regulate ion channels. *J. Gen. Physiol.* **131,** 431–438 (2008).
2. Fan, Z. & Makielski, J. C. Anionic phospholipids activate ATP-sensitive potassium channels. *J. Biol. Chem.* **272,** 5388–5395 (1997).
3. Hansen, S. B., Tao, X. & MacKinnon, R. Structural basis of PIP2 activation of the classical inward rectifier K+ channel Kir2.2. *Nature* **477,** 495–498 (2011).
4. Whorton, M. R. & MacKinnon, R. Crystal structure of the mammalian GIRK2 K + channel and gating regulation by G proteins, PIP 2, and sodium. *Cell* **147,** 199–208 (2011).
5. Whorton, M. R. & MacKinnon, R. X-ray structure of the mammalian GIRK2-βγ G-protein complex. *Nature* **498,** 190–197 (2013).
6. Zaydman, M. A. *et al.* Kv7.1 ion channels require a lipid to couple voltage sensing to pore opening. *Proc. Natl. Acad. Sci. U. S. A.* **110,** 13180–13185 (2013).
7. Valiyaveetil, F. I., Zhou, Y. & MacKinnon, R. Lipids in the structure, folding, and function of the KcsA K+ channel. *Biochemistry* **41,** 10771–10777 (2002).
8. Rosenhouse-Dantsker, A., Noskov, S., Durdagi, S., Logothetis, D. E. & Levitan, I. Identification of novel cholesterol-binding regions in Kir2 channels. *J. Biol. Chem.* **288,** 31154–31164 (2013).
9. Fürst, O., Nichols, C. G., Lamoureux, G. & Davanzo, N. Identification of a cholesterol-binding pocket in inward rectifier K+ (Kir) channels. *Biophys. J.* **107,** 2786–2796 (2014).
10. Bukiya, A. N., Durdagim, S., Noskov, S. & Rosenhouse-Dantske, A. Cholesterol up-regulates neuronal G protein-gated inwardly rectifying potassium (GIRK) channel activity in the hippocampus. *J. Biol. Chem.* **292,** 6135–6147 (2017).
11. Green, W. Surface Charges And Ion Channel Function. *Annu. Rev. Physiol.* **53,** 341–359 (1991).
12. Bell, J. E. & Miller, C. Effects of phospholipid surface charge on ion conduction in the K+ channel of sarcoplasmic reticulum. *Biophys. J.* **45,** 279–287 (1984).
13. Coronado, R. & Affolter, H. Insulation of the conduction pathway of muscle transverse tubule calcium channels from the surface charge of bilayer phospholipid. *J. Gen. Physiol.* **87,** 933–953 (1986).
14. Schmidt, D., Jiang, Q. X. & MacKinnon, R. Phospholipids and the origin of cationic

gating charges in voltage sensors. *Nature* **444,** 775–779 (2006).

15. Xu, Y., Ramu, Y. & Lu, Z. Removal of phospho-head groups of membrane lipids immobilizes voltage sensors of K+ channels. *Nature* **451,** 826–829 (2008).
16. Ramu, Y., Xu, Y. & Lu, Z. Enzymatic activation of voltage-gated potassium channels. *Nature* **442,** 696–699 (2006).
17. Jiang, Q. X. & Gonen, T. The influence of lipids on voltage-gated ion channels. *Curr. Opin. Struct. Biol.* **22,** 529–536 (2012).
18. Zheng, H., Liu, W., Anderson, L. Y. & Jiang, Q. X. Lipid-dependent gating of a voltage-gated potassium channel. *Nat. Commun.* **2,** 250 (2011).
19. Hajdú, P., Varga, Z., Pieri, C., Panyi, G. & Gáspár, R. Cholesterol modifies the gating of Kv1.3 in human T lymphocytes. *Pflugers Arch. Eur. J. Physiol.* **445,** 674–682 (2003).
20. Brohawn, S. G., Su, Z. & MacKinnon, R. Mechanosensitivity is mediated directly by the lipid membrane in TRAAK and TREK1 K+ channels. *Proc. Natl. Acad. Sci. U. S. A.* **111,** 3614–3619 (2014).
21. Dong, Y. Y. *et al.* K2P channel gating mechanisms revealed by structures of TREK-2 and a complex with Prozac. *Science* **347,** 1256–1259 (2015).
22. Yuan, C., O'Connell, R. J., Jacob, R. F., Mason, R. P. & Treistman, S. N. Regulation of the gating of BKCa channel by lipid bilayer thickness. *J. Biol. Chem.* **282,** 7276–7286 (2007).
23. Yuan, C., O'Connell, R. J., Feinberg-Zadek, P. L., Johnston, L. J. & Treistman, S. N. Bilayer thickness modulates the conductance of the BK channel in model membranes. *Biophys. J.* **86,** 3620–3633 (2004).
24. Callahan, K. M., Mondou, B., Sasseville, L., Schwartz, J.-L. & D'Avanzo, N. The influence of membrane bilayer thickness on KcsA channel activity. *Channels* **13,** 424–439 (2019).
25. Chang, H. M., Reitstetter, R., Mason, R. P. & Gruener, R. Attenuation of channel kinetics and conductance by cholesterol: An interpretation using structural stress as a unifying concept. *J. Membr. Biol.* **143,** 53–63 (1995).
26. Crowley, J. J., Treistman, S. N. & Dopico, A. M. Cholesterol antagonizes ethanol potentiation of human brain BKCa channels reconstituted into phospholipid bilayers. *Mol. Pharmacol.* **64,** 365–372 (2003).

27. Huang, J. *et al.* CHARMM36m: An improved force field for folded and intrinsically disordered proteins. *Nat. Methods* **14,** 71–73 (2016).

28. Klauda, J. B. *et al.* Update of the CHARMM All-Atom Additive Force Field for Lipids: Validation on Six Lipid Types. *J. Phys. Chem. B* **114,** 7830–7843 (2010).

29. Jorgensen, W. L., Chandrasekhar, J., Madura, J. D., Impey, R. W. & Klein, M. L. Comparison of Simple Potential Functions for Simulating Liquid Water. *J. Chem. Phys.* **79,** 926–935 (1983).

30. Hess, B., Bekker, H., Berendsen, H. J. C. & Fraaije, J. G. E. M. LINCS: A Linear Constraint Solver for molecular simulations. *J. Comput. Chem.* **18,** 1463–1472 (1997).

31. Parrinello, M. & Rahman, A. Polymorphic Transitions in Single Crystals: A New Molecular Dynamics Method. *J. Appl. Phys.* **52,** 7182–7190 (1981).

32. Nose, S. & Klein, M. L. Constant Pressure Molecular Dynamics for Molecular Systems. *Mol. Phys.* **50,** 1055–1076 (1983).

33. Nose, S. A molecular dynamics method for simulations in the canonical ensemble. *Mol. Phys.* **52,** 255–268 (1983).

34. Hoover, W. G. Canonical dynamics: Equilibrium phase-space distributions. *Phys. Rev. A* **31,** 1695–1697 (1985).

35. Steinbach, P. J. & Brooks, B. R. New spherical-cutoff methods for long-range forces in macromolecular simulation. *J. Comput. Chem.* **15,** 667–683 (1994).

36. Darden, T., York, D. & Pedersen, L. Particle Mesh Ewald: An N log(N) Method for Ewald Sums in Large Systems. *J. Chem. Phys.* **98,** 10089–10092 (1993).

37. Essmann, U. *et al.* A Smooth Particle Mesh Ewald Method. *J. Chem. Phys.* **103,** 8577–8592 (1995).

38. Bussi, G., Donadio, D. & Parrinello, M. Canonical Sampling Through Velocity Rescaling. *J. Chem. Phys.* **126,** 014101 (2007).

39. Kopec, W., Rothberg, B. S. & Groot, B. L. de. Molecular mechanism of a potassium channel gating through activation gate-selectivity filter coupling. *Nat. Commun.* **10,** 5336 (2019).

40. Smart, O. S., Neduvelil, J. G., Wang, X., Wallace, B. A. & Sansom, M. S. P. HOLE: A program for the analysis of the pore dimensions of ion channel structural models. *J. Mol. Graph.* **14,** 354–360 (1996).
