# Supplementary material for: Lipid-protein interactions modulate the conformational equilibrium of a potassium channel
Source: Nat Commun. 2020 May 1;11:2162. doi: 10.1038/s41467-020-15741-8 (PMC7195391; doi:10.1038/s41467-020-15741-8)
Supplement: Supplementary file 3 — Reporting Summary [file 41467_2020_15741_MOESM3_ESM.pdf]

nature research

Corresponding author(s): Bert de Groot

Last updated by author(s): Mar 10, 2020

Reporting Summary

Nature Research wishes to improve the reproducibility of the work that we publish. This form provides structure for consistency and transparency in reporting. For further information on Nature Research policies, see Authors & Referees and the Editorial Policy Checklist .

Statistics

For all statistical analyses, confirm that the following items are present in the figure legend, table legend, main text, or Methods section.

n/a	Confirmed	
[ ]	[x]	The exact sample size (n) for each experimental group/condition, given as a discrete number and unit of measurement
[x]	[ ]	A statement on whether measurements were taken from distinct samples or whether the same sample was measured repeatedly
[x]	[ ]	The statistical test(s) used AND whether they are one- or two-sided<br>*Only common tests should be described solely by name; describe more complex techniques in the Methods section.*
[x]	[ ]	A description of all covariates tested
[x]	[ ]	A description of any assumptions or corrections, such as tests of normality and adjustment for multiple comparisons
[ ]	[x]	A full description of the statistical parameters including central tendency (e.g. means) or other basic estimates (e.g. regression coefficient) AND variation (e.g. standard deviation) or associated estimates of uncertainty (e.g. confidence intervals)
[x]	[ ]	For null hypothesis testing, the test statistic (e.g. F, t, r) with confidence intervals, effect sizes, degrees of freedom and P value noted<br>*Give P values as exact values whenever suitable.*
[x]	[ ]	For Bayesian analysis, information on the choice of priors and Markov chain Monte Carlo settings
[x]	[ ]	For hierarchical and complex designs, identification of the appropriate level for tests and full reporting of outcomes
[x]	[ ]	Estimates of effect sizes (e.g. Cohen's d, Pearson's r), indicating how they were calculated

Our web collection on statistics for biologists contains articles on many of the points above.

Software and code

Policy information about availability of computer code

Data collection	GROMACS 5.1, CHARMM36m force field, AMBER14 force field, Slipids force field, Fortran code (Kopec et al., Nature Communications, 10, 5366, 2019), Python script
Data analysis	The python scripts to calculate bending angle, distance, and the potassium and water energy profiles are available from http://dx.doi.org/10.6084/m9.figshare.11827809.

For manuscripts utilizing custom algorithms or software that are central to the research but not yet described in published literature, software must be made available to editors/reviewers. We strongly encourage code deposition in a community repository (e.g. GitHub). See the Nature Research guidelines for submitting code & software for further information.

Data

Policy information about availability of data

All manuscripts must include a data availability statement. This statement should provide the following information, where applicable:

- Accession codes, unique identifiers, or web links for publicly available datasets
- A list of figures that have associated raw data
- A description of any restrictions on data availability

Data supporting the findings of this manuscript are available from the corresponding authors upon reasonable request. A reporting summary for this Article is available as a Supplementary Information file. The source data from MD simulations underlying Figs 2, 3B-G, 4 B, D-F, 5 C, D, F, Supplementary Tables 2-7, and Supplementary Figs. 1-6, 7B, 8-12 are provided as a Source Data File available from http://dx.doi.org/10.6084/m9.figshare.11827806

Field-specific reporting

Please select the one below that is the best fit for your research. If you are not sure, read the appropriate sections before making your selection.

- [x] Life sciences
- [ ] Behavioural & social sciences
- [ ] Ecological, evolutionary & environmental sciences

For a reference copy of the document with all sections, see nature.com/documents/nr-reporting-summary-flat.pdf

Life sciences study design

All studies must disclose on these points even when the disclosure is negative.

Sample size	We increase the sample size (the number of simulation replicates) as much as we can, to ensure that the differences between the results of different simulation systems are statistical significant according to the standard error of the mean.
Data exclusions	We do not exclude any data.
Replication	For each simulation system, we run 10-40 replicates and calculated the averages and standard errors of the mean. Some of the simulations have large uncertainty, which is discussed in the "Sampling" section in the Supplementary Material. We also used another force field to verify our conclusions. All of the replicates are conducted successfully and are included in data analyses.<br>To verify the reproducibility of the findings, we repeated the simulations using a different force field (AMBER14 for protein, Slipids for lipids). All of the conclusions found by CHARMM36m simulations are confirmed by these simulations.
Randomization	This is not relevant to our work, as we are doing computer simulations and all of the parallel simulations used the same parameters.
Blinding	This is not relevant to our work. We are doing computer simulations, and all of the parameters are set up by the simulater.

Reporting for specific materials, systems and methods

We require information from authors about some types of materials, experimental systems and methods used in many studies. Here, indicate whether each material, system or method listed is relevant to your study. If you are not sure if a list item applies to your research, read the appropriate section before selecting a response.

Materials & experimental systems

n/a	Involved in the study	
☒	☐	Antibodies
☒	☐	Eukaryotic cell lines
☒	☐	Palaeontology
☒	☐	Animals and other organisms
☒	☐	Human research participants
☒	☐	Clinical data

Methods

n/a	Involved in the study	
☒	☐	ChIP-seq
☒	☐	Flow cytometry
☒	☐	MRI-based neuroimaging
